# Human leukocyte antigen class I expression is an independent prognostic factor in advanced ovarian cancer resistant to first-line platinum chemotherapy

**DOI:** 10.1038/sj.bjc.6605315

**Published:** 2009-09-15

**Authors:** M Shehata, A Mukherjee, S Deen, A Al-Attar, L G Durrant, S Chan

**Affiliations:** 1 Academic and Clinical Department of Oncology, Nottingham University Hospitals NHS Trust – City Campus, Nottingham NG5 1PB, UK; 2 Department of Histopathology, Nottingham University Hospitals NHS Trust – QMC Campus, Nottingham NG7 2UH, UK

**Keywords:** ovarian cancer, HLA class I, platinum resistance

## Abstract

**Background:**

Loss of HLA class I is important in ovarian cancer prognosis but its role as a prognostic indicator in relation to therapy remains unproven. We studied the prognostic potential of this antigen and its significance in relation to platinum therapy.

**Methods:**

A total of 157 primary ovarian cancers were assessed for HLA class I immunohistochemically and linked to a comprehensive database of clinicopathological variables, treatment details, and platinum sensitivity.

**Results:**

Tumours expressing high levels of HLA class I had significantly improved survival (*P*=0.044). There was a 19-month difference in the median overall survival between tumours with high and low antigen expression. HLA class I antigen expression, stage, and platinum sensitivity were independently predictive of prognosis on multivariate analysis. HLA class I antigen was shown to be expressed at higher levels in patients with good overall survival in platinum-resistant patients (*P*=0.042). HLA class I significantly correlated with overall survival on multivariate analyses (*P*=0.034).

**Conclusion:**

Low-level HLA class I expression is an independent prognostic indicator of poor clinical outcome in ovarian cancer. The survival advantage of patients with platinum-resistant tumours expressing high levels of HLA class I suggests that immunotherapy may be of use in these ovarian cancers resistant to standard chemotherapy.

In the UK, ovarian cancer is the most common gynaecological cancer and is the fourth most common after cancers of the breast, large bowel, and lung, representing approximately 5% of all cancers in women. The 5-year survival rate has only improved from 30% in the period from 1986–1990 to 38% in 1996–1999 (Quinn, 2001).

Some advances have been made in the development of novel chemotherapeutic agents; however, their impact on cancer-related mortality is limited. The current standard treatment of ovarian cancer is primary surgery followed by platinum-based chemotherapy. Regimens that contained platinum salt±taxenes have been established as the standard chemotherapy in ovarian cancer through a number of clinical studies including GOG111, OV10, ICON3, and GOG132 (McGuire *et al*, 1996; Muggia *et al*, 2000; Piccart *et al*, 2000; ICON_Group, 2002). Multiple agents in a sequential approach for more prolonged treatment (GOG182-ICON5) failed to show a benefit over standard chemotherapy of a platinum salt and a taxene combination for six cycles (Bookman, 2006). The results of adding novel targeting agents such as the VEGF-targeting monoclonal antibody bevacizumab are eagerly awaited (ICON7). However, only palliative treatment is available to patients with tumours that are resistant to platinum chemotherapy. The patients have a median survival of 6 months from the time of documented resistance (Markman *et al*, 2004). There is an urgent need for novel approaches for these tumours that are resistant to platinum chemotherapy.

Although clinicopathological prognostic factors like tumour stage, grade, age, residual disease after surgical debulking, and response to chemotherapy predict prognosis in ovarian cancer; there is large individual variation in prognosis of apparently similar tumours when using these standard clinical variables (Rubin *et al*, 1999). This discrepancy may be accounted for by variations at the molecular level, or the interaction of certain factors like the immune system in the disease process. An important mechanism by which the immune system eliminates cancer cells is through a cytotoxic T lymphocyte (CTL)-based response to abnormal peptide presented in conjunction with HLA class I antigen on tumour cells (Seliger *et al*, 2000). Through a series of sequential steps, HLA class I antigen-tumour associated antigen-derived peptide complexes are formed, transported to the cell membrane, and presented to CTL. This process leads to the formation of complexes made of the peptide with both component units of the HLA molecule (*β*2-microglobulin (*β*2m) and class I heavy chain). This complex travels to the cell membrane where peptides are presented to CTL (Momburg and Tan, 2002; Bouvier, 2003).

Previous work in breast and colorectal cancers has supported this theory, with loss of HLA class I expression shown as an independent prognostic factor (Madjd *et al*, 2005; Watson *et al*, 2006). In ovarian cancer, T-cell infiltration is associated with better survival, which suggests a role for the immune system in its prognosis (Zhang *et al*, 2003). There is evidence from our group and others that HLA class I is important in ovarian cancer prognosis (Rolland *et al*, 2007; Han *et al*, 2008) but this prognostic function is not known in the context of platinum sensitivity. The purpose of this study was to study the prognostic potential of HLA class I antigen in a group of homogenously treated ovarian cancer patients with platinum chemotherapy and correlate that with their sensitivity to chemotherapy.

## Patients and methods

### Patients

This study includes 157 patients with primary ovarian cancer treated at Nottingham University Hospitals (NUH) from the year 2000–2007. The haematoxylin/eosin (H&E) sections of these tumours were reviewed by a gynae-pathologist (SD) masked to the clinical data and pathological diagnosis. Tumours were typed and graded with representative areas marked to be cored for the arrayed blocks. Clinicopathological variables for this cohort were recorded, including age at diagnosis, FIGO stage, extent of cytoreduction, tumour grade and histological subtype. Details of adjuvant treatment, disease-free survival (DFS) and overall survival were documented for all patients. Survival was calculated from the operation date until 30 June 2008 when any remaining survivors were censored. Median follow-up was 36 months. During the study period, all patients were treated by the current standard chemotherapy with either single agent carboplatin in 65 patients (41.4%) or platinum-based combination chemotherapy in 89 patients (56.7%). Of them, 79 were given carboplatin and paclitaxel. CAP was used in four patients (2.5%). Three patients (1.9%) were treated in ICON5 trial using paclitaxel, carboplatin with gemcitabine or topotecan. Another three patients were treated according to the SCOTROC trial protocol by carboplatin, docetaxel±topotecan. Platinum-resistant cases were defined as patients who progressed with first-line platinum chemotherapy during treatment or relapsed within 6 months after treatment.

### Specimens

Tissue microarrays (TMA) were constructed from prechemotherapy paraffin-embedded tumour samples. For each tumour, H&E-stained slides were first used to locate representative areas of viable tumour tissue. Needle core-biopsies (0.6 mm) from the corresponding areas on the paraffin-embedded tumour blocks were then placed at pre-specified coordinates in recipient paraffin array blocks using a manual tissue arrayer (Beecher Instruments, Sun Prarie, WI, USA). Array blocks were constructed with 89–100 cores in each recipient block. Three copies of the array were assembled using different points from the representative tumour areas. Fresh 4 *μ*m sections were obtained from each TMA block and placed on coated glass slides to allow the immunohistochemical procedures to be performed, preserving maximum tissue antigenicity.

### Immunohistochemistry

Immunohistochemical staining for HLA class I antigen expression was performed using a routine avidin/biotin peroxidase method. Tissue array sections were first deparaffinised with xylene, rehydrated through graded alcohol, and immersed in methanol containing 0.3% hydrogen peroxide for 20 min to block endogenous peroxidase activity. To retrieve antigenicity, sections were immersed in 500 ml (pH 6.0) citrate buffer and heated for 10 min in an 800 W microwave at high power, followed by 10 min at low power. Endogenous avidin/biotin binding was blocked using an avidin/biotin blocking kit (Vector Labs, Peterborough, UK). To block non-specific binding of the primary antibody, all sections were then treated with 100 *μ*l of normal swine serum (NSS) diluted 1 : 20 in Tris-buffered saline (TBS) for 15 min.

Test sections were incubated with 100 *μ*l of mouse anti-human monoclonal antibody to HLA class I heavy chain (HC-10; a gift from Prof H Ploegh, Harvard Medical School) and to the HLA class I *β*2m subunit (A0072; Dako, Ely, UK) at dilutions of 1 : 1500 and 1 : 2000 respectively. Dilutions were made in NSS/TBS, and left to incubate on the sections for 60 min at room temperature. Positive control tissue comprised whole sections of tonsil. The primary antibody was omitted from the negative control, which was left incubated in NSS/TBS.

After washing with TBS, all sections were incubated with 100 *μ*l of biotinylated goat anti-mouse/rabbit immunoglobulin (Dako) diluted 1 : 100 in NSS/TBS for 30 min. Sections were washed again in TBS and next incubated with 100 *μ*l of pre-formed streptavidin–biotin/horseradish peroxidase complex (Dako) for 60 min at room temperature. Subsequently, visualisation of HLA class I expression was achieved using 3,3′-diaminobenzidine tetrahydrochloride (Dako). Finally, sections were lightly counterstained with haematoxylin (Sigma, Gillingham, UK), dehydrated in alcohol, cleared in xylene, and mounted with distyrene, plasticizer and xylene (DPX; BDH, Poole, UK).

### Evaluation of HLA class I

To stain for the widest range of HLA class I heavy chains with a single antibody, we selected the mouse anti-human monoclonal antibody to HLA class I heavy chain. This antibody was raised to denatured HLA class I heavy chain freed from the *β*2m light-chain element of denatured native HLA class I antigen and preferentially recognises HLA-B and HLA-C on formalin-fixed, paraffin-embedded tissue (Stam *et al*, 1986). HLA class I antigens require the presence of *β*2m to function, and co-staining these tumours for *β*2m using a commercial polyclonal rabbit anti-human antibody (A0072; Dako) allowed the generation of HLA heavy-chain/*β*2m phenotypes.

We used three different copies of the TMA providing three samples from different areas of the tumour in each case. Following initial review of the staining characteristics, we scored TMAs using a semi-quantitative ‘*H*-score’ system by two experienced independent observers (MS and AM). The scoring was performed in a coded manner with observers masked to the clinical and pathological parameters of the case. To use both the staining intensity (graded 0, absent; 1, weak; 2, moderate; 3, strong) and distribution (percent cells staining) in the assessment of each core, we calculated an intensity score (percent cells staining × intensity of staining). A mean value was calculated for the three cores from each tumour to record heterogeneously stained HLA components accurately. In case of disagreement between the two assessors, and to reach a consensus on difficult cases, a third (SD) assessor was consulted. Slides were captured digitally using NanoZoomer scanner (Hamamatsu Photonics, Welwyn Garden City, UK), using a maximum magnification of × 200.

The cut-off value for positivity for either antigen was an *H* score of >10. Cell-surface expression of HLA requires the expression of both heavy chain and *β*2m. The HLA heavy chain+/*β*2m+phenotype was defined to be HLA class I positive, and any deviation from that was considered HLA down-regulation.

### Statistical analysis

Statistical analysis of the study data was performed using the SPSS package (version 15 for Windows; SPSS Inc, Chicago, IL, USA). Pearson's *χ*^2^-tests were used to determine the significance of associations between categorical variables. Overall survival calculations included all patients who died during the follow-up. Survival rates were calculated using the Kaplan–Meier method; differences between groups were tested using the log-rank test. Events for DFS and overall survival were defined as follows: time of disease relapse or death (for DFS) and time of death (for overall survival) respectively. The Cox proportional-hazards model was used for multivariate analysis to determine the relative risk and independent significance of individual factors. In all cases *P*-values <0.05 were considered as statistically significant.

Ethical approval to carry out the study was granted by the Derbyshire Local Research Ethics Committee.

## Results

### Clinicopathological characteristics

The median age at diagnosis was 61 years (range 33–87 years). The majority of cases were at advanced stages. 59% of the patients had stage 3 and 4 disease (*n*=92). A total of 114 patients (72.6%) had grade 3 tumours, 23 (14.6%) had grade 2, and 20 (12.7%) had well-differentiated tumours. Serous and endometrioid were the commonest histological subtypes in this cohort. More than 44% of cases (*n*=69) were deemed to be suboptimally debulked after initial surgery. Carboplatin alone was used in 41.4% (65 out of 157) of patients whereas combined platinum regimens were given in 56.7% (89 out of 157). Three patients (1.9%) refused chemotherapy. Table 1 shows the full clinicopathological characteristics of the patient cohort.

### HLA class I staining

**Figure 1:**
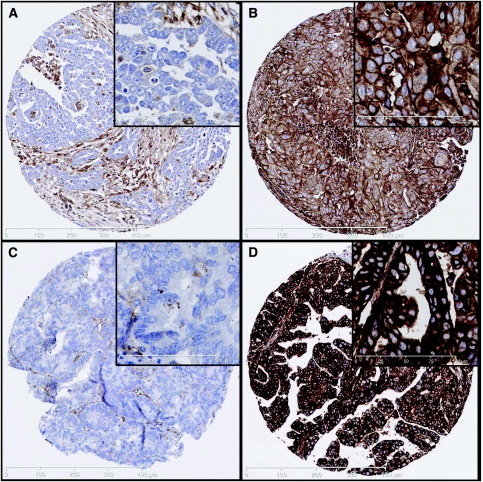
Immunohistochemical staining of tissue microarray cores with *β*2m and hC10 antibodies. (**A** and **B**) Cores from tumour showing negative (**A**) and positive (**B**) *β*2m staining. (**C** and **D**) Cores of tumour showing negative (**C**) and positive (**D**) hC10 staining. Original magnification × 100; insets × 400.

Staining was seen predominantly within the cell membrane and some cytoplasmic staining. There was no staining in negative controls. The use of *H* score allowed the heterogeneity of HLA class I expression to be taken into account. The mean *H* score was calculated by obtaining three cores from each tumour. The cut-off value for positivity for both HLA class I antigens was an *H* score of >10 and this cut-off value was previously found to separate high expression from low expression effectively (Rolland *et al*, 2007). 59% of tumours were positive for *β*2m and 63% were positive for HC10. Overall, 52% were HC10+/*β*2m+ (*n*=82) whereas 46% were negative (*n*=72). Three cores (1.9%) were unscorable due to insufficient tumour tissue left after the staining procedure. Figure 1 shows positively and negatively stained cores using the HC10 and *β*2m antibodies.

### Correlation of HLA class I expression with patient characteristics including survival

In univariate analysis, using the *χ*^2^-test, no significant relationship between HLA class I expression and the standard clinical and pathological variables was detected except in clear-cell carcinoma (CCC) and in relation to patients' age. The level of expression was significantly less in CCC subtype (*P*=0.049), and was lower in older (>60 years) patients (*P*=0.035; Table 2).

**Figure 2:**
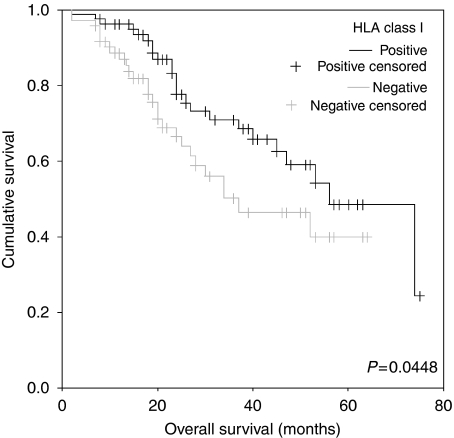
Association of HLA-I expression with overall survival time in 154 patients with primary ovarian cancer. Median survival time was 37 months for patients with negative HLA-I expression (HLA-I negative, *n*=72) compared with 56 months for patients with positive HLA-I expression (HLA class I positive, *n*=82). The difference was significant in the log-rank test (*P*=0.0448).

Correlations between HLA class I expression and patient survival were also assessed using Kaplan–Meier survival curves and log-rank testing (Figure 2). There was a statistically significant difference in the overall survival between patients with high and low HLA class I expression, with high expression predicting an improved outcome (log rank=4.03; *P*=0.045). The median overall survival was 56 and 37 months for high and low antigen expression respectively. This equates to a 19-month difference in survival between the two groups. To assess whether HLA class I status was an independent marker of prognosis, the relative influence of its expression and other known standard clinicopathological prognostic variables were included in a multivariate analysis. Factors shown to predict prognosis independently were FIGO stage, platinum sensitivity, and HLA class I expression. These factors were included in a Cox multivariate regression analysis. As shown in Table 3A, HLA class I was seen to retain its power to predict an improved prognosis in the study population, independent of other prognostic factors (HR=0.524, 95% CI=0.291–0.946; *P*=0.032).

Although progression-free survival was also worse in patients with low expression compared to patients with high expression (19 *vs* 36 months), it did not reach statistical significance (log rank=0.58, *P*=0.446).

**Figure 3:**
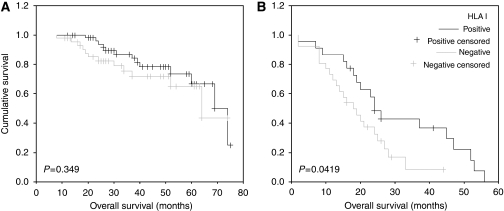
Association of HLA class I expression with overall survival time in patients with primary ovarian cancer stratified according to their platinum sensitivity. (**A**) The Kaplan–Meier survival graph in platinum-sensitive and (**B**) platinum-resistant patients. The difference in survival was only significant in the platinum-resistant group; log-rank test (*P*=0.0419).

This group of patients was stratified according to their platinum sensitivity. For both platinum-sensitive and -resistant groups, HLA class I-positive group tend to do better than HLA class I-negative group in terms of overall survival but this was only statistically significant in the platinum-resistant group (log rank=4.14; *P*=0.0419) (Figure 3). The median survival in the platinum-resistant HLA-positive and -negative groups was 24 and 18 months respectively. In the platinum-resistant group, HLA class I was the only marker that significantly correlated with overall survival (*P*=0.042). When HLA class I was included in a multivariate model that included stage, extent of debulking surgery, and grade, HLA class 1 remained statistically significant (HR=0.034, 95% CI=0.202–0.932; *P*=0.034; Table 3B).

Other clinicopathological variables that were associated with favourable overall survival in this cohort of patients were lower tumour stage (*P*<0.001) and grade (*P*=0.04), optimal debulking (*P*=0.005), and platinum sensitivity (*P*<0.001).

Sixty-five patients were found to have measurable disease before chemotherapy; three of whom were not assessed for response. Of the 62 patients remaining, the response rate (CR and PR) to single agent carboplatin was 87.5% compared to 78.9% in the combination chemotherapy group (*P*=0.412). Also, there was no statistically significant difference in chemoresponse when patients were stratified by their expression of HLA class I (*P*=0.522).

## Discussion

There is increasing evidence from animal studies that the immune system acts as an intrinsic tumour suppressor and prevents tumour growth. However, the inherent variation in tumour cell phenotype associated with either microsatellite instability or chromosomal instability may facilitate the avoidance of immune attack in a process known as ‘immune-editing’. Tumour cells which lose antigen, HLA molecules, or produce immune suppressive cytokines may thus have a selective advantage, and markers of this process can function as prognostic indicators (Dunn *et al*, 2002).

The loss of HLA class I antigens is one of the escape mechanisms found most frequently in experimental and spontaneous tumours. Tumour cells can use multiple mechanisms to, partially or completely, down-regulate the expression of HLA class I antigens. A variety of altered HLA phenotypes have been defined in human tumours, including HLA total loss, HLA haplotype loss, HLA-specific locus down-regulation, HLA allelic losses, and a combination of these phenotypes (Garrido *et al*, 1997; Garrido and Algarra, 2001). *HLA* genes control the synthesis of molecules that are in many ways the centre of the immune function mediated by T lymphocytes and natural killer (NK) cells. An increasing proportion of tumours have been found to show such alterations (Garrido *et al*, 1993). Total or selective losses of HLA class I antigens have been reported in different human tumour types (Garrido *et al*, 1997; Garrido and Algarra, 2001).

In our study, we reported the down-regulation of HLA class I antigen in ovarian cancer which in concordance with other studies (Vitale *et al*, 2005; Han *et al*, 2008) appears to be a common event. Our findings show that HLA class I heavy chain was positive in 63% and *β*2m positive in 58% of patients. Intact HLA (HC+/*β*2m+) phenotype occurred in 52% of the cases. These figures are similar to those of Vitale *et al* who also showed that HLA heavy chain was positive in 62.7% of their cases. However, our figures were slightly different from the results of the study carried out by Han *et al* (2008) who showed that HLA-HC and *β*2m were positive in 70.7 and 63.3% respectively. Multiple reasons might contribute to the differences in frequency of HLA class I in different studies. Some of them are related to the technique of immunohistochemistry such as the characteristics of monoclonal antibody used and the subjective evaluation of the staining. Other factors might be related to the difference of the patients' population in each study.

Our findings show that intact HLA phenotype confers better prognosis in terms of overall survival. This coincides with previously published results from other groups (Han *et al*, 2008) as well as from our group on a different cohort of patients (Rolland *et al*, 2007). However, Vitale *et al* (2005) did not show a survival advantage for HLA class I in ovarian cancer. This might be due to their methodology of immunostaining for HLA class I as they stained only using HC10 antibody which stains only HLA class I heavy chains (HLA-HC) but they did not stain for *β*2m which is an essential molecule for proper function of HLA class I. Overall survival was significantly worse in patients with low expression of HLA class I. Although progression-free survival was also worse in patients within this group, it did not reach statistical significance (log rank=0.58; *P*=0.446). Overall survival is often affected by several factors such as second-line chemotherapy. In addition, we noticed a difference in survival, which was only significant in the platinum-resistant patients. This is probably because platinum-sensitive patients usually have a higher response to second-line chemotherapy compared to platinum-resistant cases whose response to second-line chemotherapy is lower even with non-platinum chemotherapy (Main *et al*, 2006). Thus, these patients may have to rely on other mechanisms to battle their disease. This was shown in our series by HLA class I expression which is important for the immune system to recognise and attack cancer cells.

The previous work from our group included patients treated since 1982 and a considerable number of them were not treated by the current standard of treatment with platinum-based chemotherapy. The current study included a homogenous group of patients treated at NUH since 2000 in a uniform manner with the current standard chemotherapy. Both studies showed that HLA class I down-regulation is poor prognostic factor independent of other clinicopathological criteria. Although there was a difference in patient cohort and the treatment received, we were able to reproduce similar findings in a more representative cohort that reflects current practice of ovarian cancer management.

Because platinum-based chemotherapy was the only treatment received by the patients included in this study, we had the opportunity to correlate our findings in relation to platinum sensitivity which is a major determinant of prognosis in ovarian cancer. This has not been reported before from any other group. From our data, patients with platinum-sensitive tumours had significantly better survival compared to platinum-resistant tumours with a median survival of 74 and 21 months in the two groups respectively (log rank=68.24; *P*<0.001). There is currently no effective treatment for patients with platinum-resistant disease.

In the Health Technology Assessment of three drugs used in second-line treatment of ovarian cancer, it has been shown that in patients with platinum-resistant disease there was a low probability of response to treatment with pegylated liposomal doxorubicin hydrochloride, topotecan, or paclitaxel. Response rates varied from 6.7 to 13.3%. Likewise, there was little difference between the three comparators in relation to overall survival, with median survival times varying from 36.7 to 54.3 weeks (Main *et al*, 2006). This underscores the need for novel therapeutic approaches in such a group of patients.

In our study, there were no significant difference about type of treatment (single or combination) and number of cycles received between HLA class I-positive and -negative cases. We found that HLA class I down-regulation is associated with poor overall survival in platinum-sensitive and -resistant groups of patients but this reached statistical significance only in the resistant group. This suggests that in the absence of effective chemotherapy for the platinum-resistant group, the immune system might be important in the prognosis of that particular group of patients and hence should be a target for cancer vaccines.

Alternatively, a role for NK cells in the immune-mediated response to a tumour could be suggested in case of down-regulation of HLA class I. When HLA class I antigen expression is lost, killer cell inhibitory receptors on the surface of NK cells (which produce an inhibitory signal when bound to HLA class I antigen) no longer function. This could result in increase in NK cell activity and might help to compensate for the decrease in T-cell killing associated with down-regulation of HLA class I (Ljunggren and Karre, 1990). However this theoretical advantage of NK cells was not supported by our data. However, it was previously reported that a broad spectrum of tumours often up-regulates the expression of non-classical HLA class I, like HLA-G which dampens the NK-cell responses by engaging inhibitory receptors ILT-2 and KIR2DL4 (Rouas-Freiss *et al*, 2005; Urosevic and Dummer, 2008). This was also seen in ovarian cancer where HLA-G was not only expressed in high-grade serous carcinoma but also conferred tumour cells aggressiveness (Seliger *et al*, 2003). These findings suggest that HLA-G expression represents a mechanism of resistance of NK cells in a manner that parallels its mechanism to protect against NK-mediated foetal tissue rejection in the placenta (Hunt *et al*, 2006).

Clear-cell carcinoma represents a special subtype of ovarian cancer with known bad prognosis and resistance to platinum chemotherapy (Sugiyama *et al*, 2000). We found a significant correlation between HLA class I down-regulation and clear-cell subtype compared to other histological subtypes. As expected, no significant difference in survival was noted in this group when compared to serous carcinoma. This is because cases of CCC are diagnosed, in general, as early stage. The outcome of such patients does not differ from that of serous adenocarcinoma.

Our report shows that down-regulation of HLA class I is a poor prognostic factor in ovarian cancer independent from other clinicopathological markers. The HLA class I-negative status represents a class of patients who do worse irrespective of current therapy. The survival advantage of patients with platinum-resistant tumours expressing high levels of HLA class I suggests that immunotherapy might be important as a therapeutic option in the management of these patients.

## Figures and Tables

**Table 1:** Patients' clinicopathological characteristics and HLA-I frequencies in all patients (A), and in platinum resistant patients (B)

**Characteristics**	**Frequencies**	**Percentages**
*(A)*		
*Pathology*		
Serous	88	56.1
Mucinous	12	7.6
Endometrioid	33	21
Clear cell	21	13.4
Other	3	1.9
		
*Grade*		
1	20	12.7
2	23	14.6
3	114	72.6
		
*Debulking surgery*		
Optimal	88	56.1
Suboptimal	69	43.9
		
*Stage*		
IC	44	28
II	21	13.4
III	71	45.2
IV	21	13.4
		
*Chemotherapy*		
Carboplatin monotherapy	65	41.4
Carboplatin combination	89	56.6
No chemotherapy	3	1.9
		
*Platinum sensitivity*		
Sensitive	104	66.2
Resistant	50	31.8
Unknown	3	1.9
		
*HLA class I*		
Positive	82	58.6
Negative	72	45.9
Unknown	3	1.9
		
*hC-10*		
Positive	99	63.1
Negative	56	35.7
Unknown	2	1.3
		
*β2m*		
Positive	92	58.6
Negative	63	40.1
Unknown	2	1.3
		
*Relapse status*		
Relapsed	75	47.8
Relapse free	75	47.8
Unknown	7	4.5
		
*Survival status*		
Living	103	65.6
Dead	54	34.4
		
*(B)*		
*Pathology*		
Serous	38	74.5
Mucinous	1	2
Endometrioid	6	11.8
Clear cell	4	7.8
Other	2	3.8
		
*Grade*		
1	3	5.9
2	5	9.8
3	43	84.3
		
*Debulking surgery*		
Optimal	16	31.4
Suboptimal	35	68.6
		
*Stage*		
IC	2	3.9
II	4	7.8
III	30	58.8
IV	15	29.4
		
*Chemotherapy*		
Carboplatin monotherapy	14	27.5
Carboplatin combination	37	72.5
No chemotherapy	0	0
		
*HLA class I*		
Positive	22	43.1
Negative	27	52.9
Unknown	2	3.9
		
*HC-10*		
Positive	29	56.9
Negative	20	39.2
Unknown	2	3.9
		
*β2m*		
Positive	27	52.9
Negative	23	45.1
Unknown	1	2
		
*Relapse status*		
Relapsed	49	96.1
Relapse free	0	0
Unknown	2	3.9
		
*Survival status*		
Living	7	13.7
Dead	44	86.3

**Table 2:** Tumour expression of HLA-I *vs* clinicopathological features

**Variables**	**HLA-I +ve**	**HLA-I −ve**	***P*-value**
*Age groups*			
>60 Years	62	38	**0.035**
⩽60 Years	45	55	
			
*Pathology*			
Serous	51	34	0.078
Mucinous	6	6	
Endometrioid	18	15	
Clear cell	7	14	
Other	3		
			
*CCC vs others*			
CCC	7	14	**0.049**
Other types	75	58	
			
*Stage*			
I and II	35	30	0.899
III and IV	47	42	
			
*Grade*			
1 and 2	28	15	0.264
3	64	48	
			
*Debulking surgery*			
Optimal	49	39	0.484
Suboptimal	33	33	
			
*Platinum sensitivity*			
Sensitive	59	44	0.189
Resistant	22	26	

Abbreviation: CCC=clear-cell carcinoma.

The association of HLA-I with CCC is also shown compared to the other pathological types combined. Significant *P*-values are shown in bold.

**Table 3:** Multivariate analysis of the effects of investigated parameters on overall survival using a Cox proportional-hazard regression model in all patients (A) and the platinum-resistant group (B)

		**95% CI for HR**		
**Variables**	**HR**	**Lower**	**Upper**	***P*-value**
*(A)*				
*Grade*				
3	1			
1+2	0.997	0.478	2.080	0.994
				
*Stage*				
I+II	1			
II+IV	2.787	1.116	6.963	**0.028**
				
*Debulking surgery*				
Suboptimal	1			
Optimal	0.968	0.520	1.804	0.920
				
*Platinum sensitivity*				
Sensitive	1			
Resistant	6.129	3.055	12.297	**<0.001**
				
*HLA class I*				
Negative	1			
Positive	0.524	0.291	0.946	**0.032**
				
*(B)*				
*Grade*				
3	1			
1+2	0.659	0.273	1.590	0.323
				
*Stage*				
I+II	1			
II+IV	1.094	.357	3.348	0.875
				
*Debulking surgery*				
Suboptimal	1			
Optimal	0.792	0.381	1.646	0.531
				
*HLA class I*				
Negative	1			
Positive	0.440	0.206	0.938	**0.034**

The prognostic impact of HLA class I was adjusted for well-established clinical prognostic factors including histological grade, FIGO stage, debulking surgery and platinum sensitivity. Significant *P*-values are shown in bold. Platinum sensitivity is not included in B as the data included the platinum-resistant patients only.
